# Psychosocial encounters correlates with higher patient-reported functional quality of life in gynecological cancer patients receiving radiotherapy

**DOI:** 10.1186/s13014-015-0339-2

**Published:** 2015-02-06

**Authors:** Penny Fang, Kay See Tan, Surbhi Grover, Mary K McFadien, Andrea B Troxel, Lilie Lin

**Affiliations:** Department of Biostatistics and Epidemiology, University of Pennsylvania, PCAM/TRC 4 West, 3400 Civic Center Blvd, Philadelphia, PA 19104 USA; Department of Radiation Oncology, Perelman School of Medicine of the University of Pennsylvania, Philadelphia, PA USA

**Keywords:** Gynecologic cancer, Radiation therapy, Patient reported outcomes, FACT-G, MDASI, Psychological support

## Abstract

**Background:**

Our objective was to assess longitudinal health-related quality of life (HRQoL) in patients treated with radiotherapy for gynecologic malignancy and assess the relationship of psychosocial encounters on HRQoL.

**Methods:**

Women with gynecologic malignancy were prospectively enrolled and HRQoL assessed before, during, and after radiotherapy treatment using validated measures. Treatment and demographic information were reviewed. Mixed-effects models were used to assess changes in quality of life (QoL) over time and association of psychologist and social worker encounters with overall QoL as well as subdomains of QoL.

**Results:**

Fifty-two women were enrolled and 41 completed at least one assessment. Fatigue (p = 0.008), nausea (p = 0.001), feeling ill (p = 0.007), and being bothered by side effects (p < 0.001) worsened on treatment with subsequent improvement. By follow-up, patients reported increased functional well-being (FWB) with significant decrease in worry (p = 0.003), increase in enjoyment of things usually done for fun (p = 0.003) and increase in contentment (p = 0.047). Twenty-three patients had at least one interaction with a social worker or psychologist during treatment. Each additional interaction was associated with a 2.12 increase in FWB score from before to after treatment (p = 0.002), and 1.74 increase from on to after treatment (p = 0.011). Additional interactions were not significantly associated with changes in overall FACT score (p = 0.056) or SWB (p = 0.305).

**Conclusions:**

Patient-reported HRQoL significantly worsened during radiotherapy treatment with subsequent improvement, affirming transiency of treatment-induced toxicities. Our preliminary study suggests that clinically-recommended psychological and social work interventions have potential value with respect to improving patient QoL during radiotherapy. Larger studies are needed to validate our findings.

## Introduction

As gynecological cancer patients live longer due to improved therapies, studies have reported that many survivors experience significant long-term sequelae from their cancer and treatment that can significantly impact their health related quality of life (HRQoL) [1,2]. HRQoL has been found to significantly affect patient’s survivorship experiences, adherence to subsequent treatment, and even predict survival after controlling for demographic and clinical characteristics [3,4]. Capturing patients’ macro state of well-being through HRQoL is also important to better and more holistically measure and understand the effectiveness of treatment beyond patient survival.

Treatment of gynecologic malignancies often involves a multi-modality approach and is associated with high morbidity and changes in bowel, bladder, hormonal, sexual, and reproductive function, many of which can cause emotional and psychosocial in addition to physical distress [1,5]. Poor psychosocial adjustment in patients with gynecologic cancer has been associated with radiotherapy in particular, as well as with multi-modality treatment, younger patient age, longer treatment duration, disengaged coping style, and inadequate social support [1].

QoL of gynecologic cancer patients tends to be at its worst between time of diagnosis and completion of radiotherapy [1]. When patients treated for gynecologic cancer were surveyed and asked to rate their emotional distress while on treatment, 57% reported needing help dealing with emotional problems, suggesting a high prevalence of potential need for additional psychosocial support [6]. The duration between initial diagnosis and on-treatment is also the time when oncologists interact most with the patient, and provides the greatest opportunity for intervention. Therefore, it is important to study the emotional and functional components of QoL in gynecologic cancer patients while on active treatment, a critical window for additional clinician support. Previous studies suggest that psychosocial interventions for cancer patients in the acute setting improve patients’ psychological well-being [7-9]. Because radiation therapy has been associated with risk of poor adjustment, our objective was to identify the trajectory of patients’ quality of life (QoL) during and after radiotherapy, particularly the role of psychosocial interactions on patient QoL.

Characterization of gynecologic cancer patient-reported QoL over the treatment course may more effectively and appropriately identify patients with substantial emotional support needs who may benefit most from a social worker or psychologist referral. This study assesses longitudinal QoL and investigates psychosocial encounters as a predictor for improved QoL among patients with gynecologic cancer receiving radiation therapy. We hypothesized that psychosocial encounters would improve QoL during and after radiation therapy.

## Methods/Materials

### Study population

Women with gynecologic malignancies undergoing radiation therapy (RT) with or without systemic therapy were prospectively enrolled in an IRB approved prospective clinical study to assess HRQoL between 2009 and 2012 at University of Pennsylvania (PENN). This QOL study was open to all patients receiving radiotherapy at PENN regardless of primary disease site as long as the treatment was with definitive curative intent. Patients were recruited either at the time of consultation or at the time of simulation for radiotherapy treatment planning. The protocol for data collection, storage and retrieval complied with the PENN Institutional Review Board and Health Insurance Privacy and Portability Act regulations. QoL assessments were collected pre-RT, during RT, and within 3 months post-RT using MD Anderson Symptom Inventory (MDASI) and Functional Assessment of Cancer Therapy-General (FACT-G). Eligibility criteria included age 18 and above and ability to read and understand English. Patient-reported QoL assessments were matched and compared with specific, corresponding outcomes extracted from physician encounter notes from corresponding time points. Diagnosis, treatment modality, concurrent chemotherapy treatment and demographics were also reviewed.

### Measures of QoL

HRQoL surveys were obtained at baseline, during, and approximately 3 months after RT using the following validated measures:

#### MD Anderson Symptom Inventory (MDASI)

The MDASI is a 26-item questionnaire assessing core symptom severity and interference with life activities. In the first part, patients rate the severity of symptoms experienced in the last 24 hours on a 0–10 scale with 0 being not present and 10 being “as bad as you can imagine”. The second part entails rating how symptoms have interfered with various life activities, mood, and life enjoyment on a 0–10 scale, with 0 being “did not interfere” and 10 being “interfered completely”. Due to the larger number of items included in the MDASI, we focused on selected MDASI core items based on commonly occurring symptoms among patients in this population, namely pain, fatigue, disturbed sleep, distressed, difficulty remembering things, lack of appetite, sadness and drowsiness.

#### Functional Assessment of Cancer Therapy-General (FACT-G)

The FACT-G was developed for cancer patients with cancer at various sites, and its reliability and validity has been reported [10]. FACT-G is scored by aggregating the individual scale scores, with higher scores indicating better quality of life. The FACT-G is a commonly used tool measuring general quality of life across four subscales: physical well-being (7 items), social/family well-being (7 items), emotional well-being (6 items), and functional well-being (7 items). The patient is asked to grade their response as it applies to the past 7 days on 5 levels including: 0-not at all, 1-a little bit, 2-somewhat, 3-quite a bit, and 4-very much. Physical well-being (PWB) includes lack of energy, nausea, trouble meeting needs of family, pain, being bothered by side effects of treatment, feeling ill, and being forced to spend time in bed. Social/family well-being (SWB) includes feeling close to and getting emotional support from family and/or friends, being satisfied with family communication about the patient’s illness, satisfaction with sex life, and feeling close to a partner or main support. Emotional well-being (EWB) includes feeling sad, being satisfied with coping, losing hope, feeling nervous, worrying about dying, and worrying about worsening condition. Functional well-being (FWB) includes being able to work (including work at home) and find work fulfilling, enjoying life, accepting illness, sleeping well, enjoying things usually done for fun, and being content with QoL. Each FACT-G subscale is independently scored (on a scale of 0–28, except EWB 0–24) and also contributes to a total FACT-G cumulative score (scale of 0–108, higher score indicates better QoL).

### Medical and demographic characteristics

Clinical data were abstracted from patients’ electronic medical records. Other data collected included patient age, race, ethnicity, cancer type, stage at diagnosis, radiation technique and dose, history of prior radiation therapy, and receipt of systemic therapy. Psychosocial encounters were grouped as psychosocial support, financial resource assistance, cancer center counseling, or allied health coordination (including lodging/transportation/insurance/employment) by reviewing the patient medical records.

### Statistical analyses

Descriptive statistics of HRQoL and medical factors were summarized for study participants. Comparisons between the subjects included and excluded from the final analyses were performed using the Wilcoxon rank sum test for continuous variables and Fisher’s exact test for categorical variables. Mixed-effects models with patient random effect including adjustment for time were used to analyze change in patient-reported QoL and symptoms over the treatment course. Time (pre-, on-, post-RT) was treated as categorical. Mixed-effects models were also used to analyze effect of number of encounters with psychologists and social workers on changes in functional quality of life. These models were adjusted for patient age, malignancy type, and body mass index. All mixed-effects models assume correlation between all measurements on the same subject, and that the correlation was the same for all pairs of time points. All statistical analyses were conducted using SAS 9.3 (SAS Institute, Cary, NC) with a two-sided Type I error rate of 0.05.

## Results

### Patient demographics

Fifty-two gynecologic cancer patients were enrolled, of whom 41 patients completed at least one QoL assessment. Eleven patients were enrolled but did not have an initial evaluation or complete subsequent assessments and were excluded from subsequent analyses. Comparisons between included (N = 41) and excluded (N = 11) subjects indicated that the two groups did not differ in terms of demographic characteristics such as age (p = 0.771) and BMI (p = 0.638) or clinical characteristics such as stage (p = 0.192), radiation technique (p = 0.198), and cancer type (p = 0.449). Of 41 patients included in the analyses, 25 patients had endometrial cancer, 14 had cervical cancer, 1 had vaginal cancer, and 1 had vulvar cancer (Table 1). The median patient age was 61 (range, 33–74). At diagnosis, 58.3% of patients were stage I, 19.4% stage II, 19.4% stage III, and 3% stage IV. Twenty-four patients had chemotherapy, either sandwich or concurrently with RT. For RT treatment, 10 patients received IMRT only, 12 received brachytherapy only, and 19 received both IMRT and brachytherapy. None of the patients had history of prior radiation therapy for gynecologic malignancy.

**Table 1:** **Patient characteristics**

	**n (%)**
Number of patients completed assessments/included	41
Median age at study entry, y (range)	61 (33–74)
Cancer type	
Endometrial	25 (61.0)
Cervical	14 (34.2)24
Vaginal	1 (2.4)1
Vulvur	1 (2.4)1
**Stage at diagnosis, n (%)**	
0	0
I	21 (58.3)
II	7 (19.4)
III	7 (19.4)
IV	1 (3.0)
N/A	5
**Radiation technique, n (%)**	
IMRT	10 (24.4)
Brachytherapy	12 (29.3)
Both IMRT and Brachytherapy	19 (46.3)
**Systemic therapy, n (%)**	
None	17 (41.5)
Chemotherapy	24 (58.5)
Previous radiation therapy	0
**BMI at baseline, n (%)**	
<25	12 (29.3)
25-29	10 (24.4)
≥30	19 (46.3)
**Employed within last 6 months**	
No	23 (56.1)
Yes	18 (43.9)
**Marital status**	
Single/divorced/separated/widowed	13 (34.2)
Married	25 (65.8)

### Predictors of baseline or pre-treatment QoL

Age, employment status, marital status, ECOG, cancer type, chemotherapy, and cancer stage were not predictive of baseline FACT-G QoL scores at the 0.05 significance level.

### Changes in patient-reported symptoms over time

**Figure 1:**
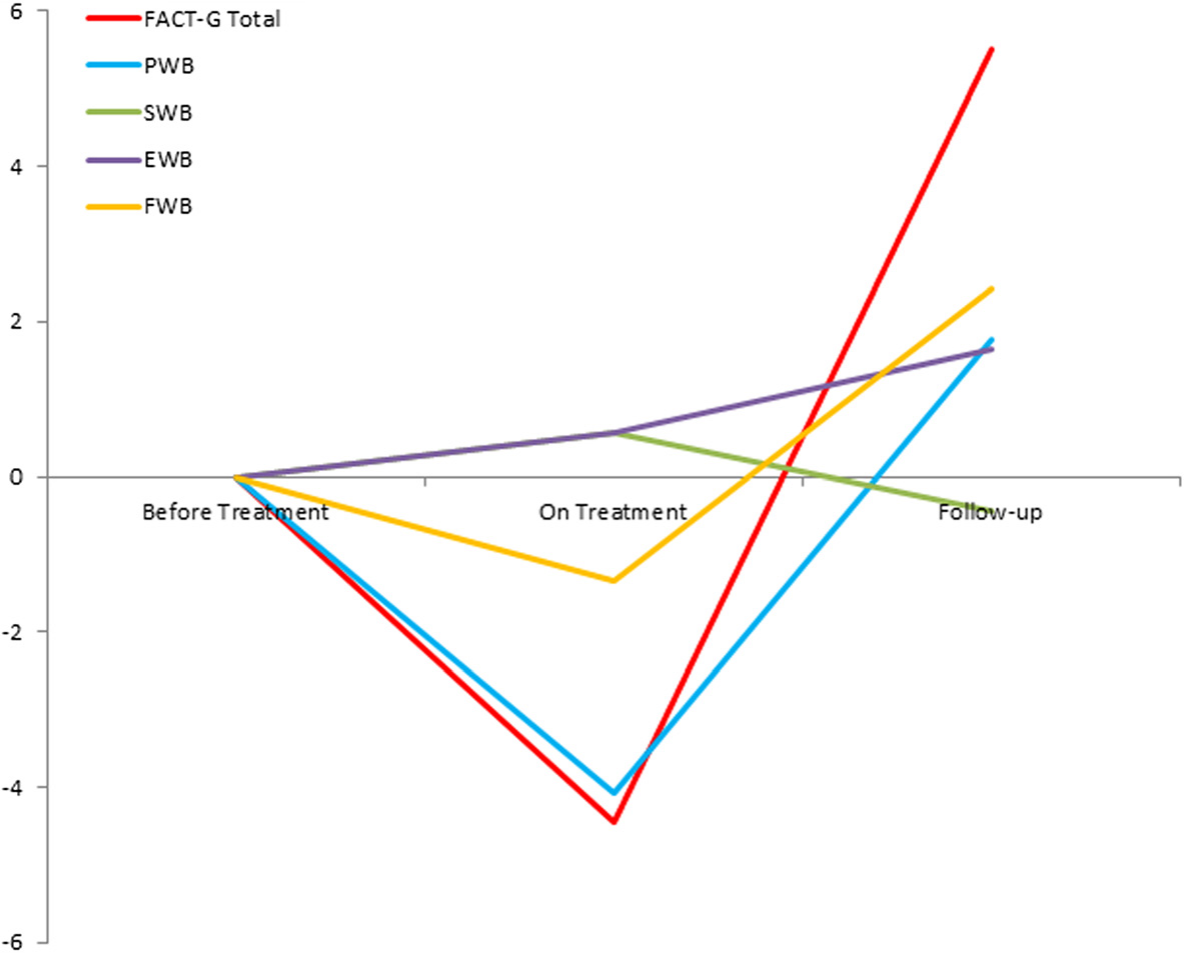
**Change in gynecologic cancer patient Functional Assessment of Cancer Therapy-General (FACT-G) quality of life scores over the treatment course, as compared to pre-treatment score.**

Table 2 presents the mean scores of FACT-G subscales and selected MDASI core items at each assessment time point in aggregate and by treatment modality. Overall, the changes in scores over time were significant in FACT-G total (p = 0.006), PWB (p < 0.001) and FWB (p = 0.004) (Figure 1). Specifically, there were significant improvements from on-RT to follow-up FACT-G total (slope, 9.93; p = 0.001), PWB (5.86; p < 0.001) and FWB (3.75; p = 0.001). PWB significantly worsened from pre-RT to on-RT (−4.07, p < 0.001), while FWB significantly increased between pre-RT and follow-up (2.41, p = 0.026). FACT-G patient-reported fatigue increased from before to on-treatment (p = 0.008) and subsequently decreased after treatment (p = 0.009). Patient-reported nausea (p = 0.001), feeling ill (p = 0.007), and being bothered by side effects of treatment (p < 0.001) on the FACT-G all increased on treatment and decreased after treatment. However, patients reported “having increased trouble meeting the needs of their families” from on-treatment to follow-up on the FACT-G (p = 0.012).

**Table 2:** **Summary of FACT-G and MDASI scores**

**Radiation technique**						
	**Total (N = 41)**		**IMRT+/−brachy (N = 29)**		**Brachy (N = 12)**	
	**N**	**Mean (SD)**	**N**	**Mean (SD)**	**N**	**Mean (SD)**
FACT-G total and subscale scores*						
Total FACT-G						
Pre	33	80.4 (20.8)	23	81.1 (20.0)	10	78.6 (23.7)
On	30	78.5 (22.2)	22	77.4 (23.0)	8	81.4 (21.0)
Post	27	84.7 (17.6)	21	86.9 (17.5)	6	77.1 (17.5)
Physical well-being						
Pre	33	21.4 (6.9)	23	21.5 (6.6)	10	21.3 (7.9)
On	30	18.3 (8.1)	22	17.6 (8.5)	8	20.2 (6.8)
Post	27	22.5 (5.9)	21	23.4 (5.3)	6	19.5 (7.4)
Social well-being						
Pre	33	23.6 (4.4)	23	23.8 (4.5)	10	23.0 (4.3)
On	30	24.4 (4.5)	22	24.1 (4.9)	8	25.3 (3.1)
Post	27	23.2 (5.9)	21	23.3 (6.4)	6	22.8 (4.2)
Emotional well-being						
Pre	33	17.4 (5.4)	23	17.8 (5.5)	10	16.5 (5.1)
On	30	18.1 (5.4)	22	18.2 (5.5)	8	17.9 (5.7)
Post	27	19.1 (3.9)	21	19.3 (3.9)	6	18.5 (4.1)
Functional well-being						
Pre	33	18.0 (6.7)	23	18.0 (6.5)	10	17.8 (7.5)
On	30	17.6 (7.6)	22	17.5 (7.9)	8	18.0 (7.5)
Post	27	19.9 (7.0)	21	20.9 (7.0)	6	16.3 (6.3)
Selected MDASI core-items**						
Pain						
Pre	32	1.5 (2.7)	22	1.4 (2.7)	10	1.8 (2.6)
On	28	1.7 (2.5)	21	2.1 (2.8)	7	0.4 (0.8)
Post	27	1.1 (2.3)	21	0.7 (1.2)	6	2.8 (4.2)
Fatigue						
Pre	33	2.2 (2.5)	23	1.6 (1.9)	10	3.5 (3.2)
On	29	3.9 (2.6)	21	4.2 (2.8)	8	3.0 (2.1)
Post	27	3.3 (3.2)	21	2.7 (2.9)	6	5.5 (3.7)
Sleep						
Pre	33	2.5 (3.1)	23	2.3 (2.8)	10	3.0 (3.9)
On	28	2.4 (2.8)	20	2.6 (2.9)	8	1.8 (2.8)
Post	27	2.9 (3.3)	21	2.5 (3.0)	6	4.0 (4.4)
Upset						
Pre	33	3.0 (3.0)	23	3.0 (2.9)	10	2.9 (3.5)
On	28	2.1 (2.8)	20	2.3 (3.2)	8	1.8 (1.7)
Post	27	2.7 (3.4)	21	2.4 (3.1)	6	3.5 (4.4)
Memory						
Pre	31	1.7 (2.7)	21	1.4 (2.2)	10	2.3 (3.7)
On	29	1.1 (1.2)	21	1.1 (1.3)	8	1.1 (1.1)
Post	26	2.2 (2.5)	20	2.1 (2.3)	6	2.5 (3.2)
Appetite						
Pre	33	1.3 (2.5)	23	0.9 (1.6)	10	2.2 (3.7)
On	29	1.8 (2.2)	21	1.9 (2.3)	8	1.6 (1.9)
Post	27	1.2 (2)	21	0.9 (1.7)	6	2.5 (2.6)
Sad						
Pre	33	2.8 (3.5)	23	2.7 (3.5)	10	3.0 (3.7)
On	29	2.1 (2.6)	21	2.2 (2.7)	8	1.9 (2.6)
Post	27	2.3 (2.5)	21	2.0 (2.3)	6	3.3 (3.1)
Drowsy						
Pre	33	1.5 (2.4)	23	0.9 (1.3)	10	2.7 (3.6)
On	29	2.8 (2.8)	21	2.9 (2.9)	8	2.5 (2.6)
Post	27	2.7 (3.1)	21	2.4 (2.7)	6	3.7 (4.2)

*FACT-G: higher score implies higher level of HRQoL.

**MDASI: lower score implies higher level HRQoL.

From pre-treatment to follow-up, patients reported a significant decrease in worry that their condition will worsen (p = 0.003), increase in enjoyment of things usually done for fun (p = 0.003) and increase in contentment with quality of life on the FACT-G (p = 0.047). Patients report an increased ability to enjoy their lives after treatment compared to when they were on treatment on the FACT-G (p = 0.012).

Significant changes in MDASI patient-reported symptoms were fatigue (p = 0.002) and drowsiness (p = 0.006), which both increased during treatment but did not significantly decrease by follow up.

### Effect of patient interaction with social workers or psychologists on change in quality of life over time

**Figure 2:**
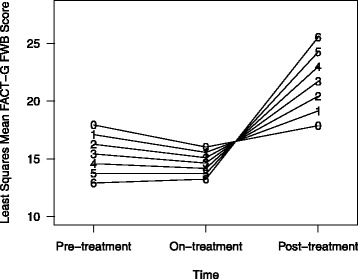
**Improved trajectory of FACT-G Functional Well-Being (FWB) from Pre/On/Post treatment with greater number of interactions with social workers and psychiatrists (shown here are estimated FWB scores for patients with median age 61, BMI <25 with cervical cancer).**

Twenty-three of the 41 patients had at least one interaction with a social worker or psychologist during radiation therapy. Total number of interactions ranged from one to six (median = 1). Thirteen patients had 1 interaction, 3 patients had two visits, 2 patients had 3 visits, and 5 patients had 4 or more encounters. Encounters included social/emotional support (27), financial resource assistance (5), cancer center counseling (4), allied health coordination/lodging/transportation/employment issues (11). Patient interaction with social workers and psychologists was predictive of improved patient functional quality of life post treatment compared to pre- and on-treatment as measured by the functional well-being subscore (Table 3). After adjusting for age, cancer type and BMI, each additional interaction with a social worker or psychologist was associated with an increase of 2.12 points in the FWB score from before to after treatment (p = 0.002), and an increase of 1.74 points from on to post-RT (p = 0.011). Additional interactions were not significantly associated with changes in overall FACT score (p = 0.056), or other subscores (SWB (p = 0.305), PWB (p = 0.271), EWB (p = 0.569). Figure 2 displays an example of the trajectory of FACT-G FWB for patients (with median age of 61, BMI < 25 with cervical cancer) from pre- to post-RT by the number of interactions with social workers and psychologists derived from the model-based estimates of the mixed-effects model.

**Table 3:** **Mixed-effects model: effects of psychosocial encounters and time on Functional well-Being (FWB)**

	**F value**	***df*** **[Num, Den]**	**Estimate**
Intercept			22.712**
Time	1.68	[2,45]	
Pre			[Reference]
On			−1.918
Post			−0.065
Psychosocial	0.00	[1,34]	−0.838
Psychosocial *Time	5.46**	[2,45]	
Psychosocial × Pre			[Reference]
Psychosocial × On			0.376
Psychosocial × Post			2.115**
Age	0.60	[1,34]	−0.078
Cancer type	2.28	[2,34]	
Cervical			[Reference]
Endometrial			4.402
Vaginal/vulvar			−3.744
BMI	3.24*	[2,34]	
BMI < 25			[Reference]
25 < =BMI < 30			−6.365*
30 < =BMI			−0.227

*P < = 0.05.

**P < 0.01.

## Discussion

Our analysis found that gynecologic cancer patients at our institution reported significant improvement in psychological QoL measures as measured by FWB subscore from before treatment to follow-up. Patients reported decreased worry in their condition worsening, increase in enjoyment of things usually done for fun, and increase in contentment with quality of life. These positive psychological changes could be due to either treatment effect or effective counseling by the physician, and thus are important for adequate valuation of HRQoL for future cost-utility analyses and treatment effectiveness analyses capturing the macro state of patient health.

Our analysis examined the impact of one particular type of intervention that could have contributed to the positive psychological changes in our patients: clinician-recommended patient encounters with social workers and psychologists. Interestingly, we found that each additional interaction with a social worker or psychologist was associated with a 2.12 point increase in functional well-being subscore from before to after treatment (p = 0.002). The functional well-being subscore includes “enjoyment of things usually done for fun”, and “increase in contentment with quality of life”, two of three psychological QoL measures that we previously identified as significantly improved in our patients over the course of study. No study to our knowledge has previously validated the benefit of psychological and social work intervention on quality of life specifically in gynecological cancer patients undergoing radiation therapy. Our study suggests that these types of interventions may indeed increase value with respect to patient quality of life during radiotherapy.

There are inherent limitations to our study including the limited number of study patients and heterogeneity of sites of malignancy (Table 1), oncologic treatments (brachytherapy, external beam radiotherapy) as well as in the types of psychosocial interactions. Also, although we accounted for marital status, we did not have comprehensive information regarding support systems at home which may be a confounding factor. Additionally, this study was not designed or powered to establish a causal relationship between number of psychosocial interactions and quality of life. We recognize the potential inflation of Type I error or false-positive results due to multiple testing and the possibility of spurious associations from the study from the many comparisons made. As noted, this set of analyses is part of a larger study intended as hypothesis-generating and requires validation in larger studies specifically designed to evaluate the impact of psychosocial encounters on quality of life in gynecologic cancer patients. Attention towards the impact of psychosocial distress on quality of life has been increasingly recognized as emerging research suggests that methods that screen for and address distress improves quality of life, but may also be associated with improved cancer outcomes [11,12]. The American College of Surgeons Commission on Cancer beginning in 2015 will require that cancer centers implement screening programs for psychosocial distress as a requirement for accreditation [13].

Patient-reported HRQoL may be useful for identifying patients who can benefit from additional support before and during treatment. We found that patient-reported HRQoL significantly decreased while on treatment, but subsequent improvement of nearly all measures occurred in the period from end of treatment to follow-up. Therefore, our study using patient-reported QoL supports the transiency of acute radiotherapy toxicity. However, prior studies have identified treatment with radiotherapy as a significant risk factor for maladjustment in gynecologic cancer patients, particularly cervical cancer patients, exacerbated by increased length of treatment, younger patient age, and greater treatment toxicity [1,14-16]. Greimel et al. found that during active treatment, gynecologic cancer patients had significantly worse QoL particularly with respect to physical and role function compared with breast cancer patients [17]. Therefore, although acute toxicities are temporary, they tend to be more severe for gynecologic patients compared with breast cancer patients. Particularly for gynecologic patients receiving radiotherapy, additional psychosocial and emotional support before and during treatment may be warranted [18].

Recent studies suggest that psychosocial interventions can be effective in improving QoL during radiotherapy. A recent randomized controlled trial compared 131 patients with advanced cancer actively undergoing RT receiving either standard care or a structured, multidisciplinary intervention involving six 90-minute sessions led by a clinical psychologist or psychiatrist addressing 5 domains of QoL: emotional, social, cognitive, physical, and spiritual [8]. Overall QoL of patients who received the intervention was significantly higher than that of the control arm as assessed by FACT-G at week 4 (p = 0.02). Patients were able to maintain their baseline QoL score as opposed to those in the control arm in whom QoL decreased [8]. A social work component in structured interventions for patients with advanced cancer improved scores in the social domain of QoL, contributing to clinically meaningful overall QoL improvement (p = 0.03) [9]. Wenzel et al. reported finding substantial patient interest in a counseling program to discuss psychosocial issues [19]. Of the patients interviewed 5–10 years after cervical cancer diagnosis, 69% stated they would have participated in a psychosocial counseling program had it been offered during their initial treatment [19].

Within the cancer center at the University of Pennsylvania, we have a dedicated team of mental health professionals trained to address the psychosocial needs of cancer patients. Referrals are typically made at patients’ request or initiated by nurses or physicians on the basis of clinical assessment while they are receiving radiotherapy. An initial comprehensive assessment of the patients’ social, emotional, physical, psychological, and financial needs is completed either via telephone or in person. Needs are prioritized and resources are explored, which include emotional support/psychological counseling, transportation to and from treatments, temporary lodging, clarification of insurance benefits, accessing unemployment and disability coverage, obtaining durable medical equipment, prescription payment assistance and accessing financial supports. Social workers partner with local and national programs to connect patients with programs that will assist with these various needs. Patients can also access counseling and management of mental health medications through referrals to both community-based mental health professionals and the Abramson Cancer Center’s cancer counseling service, which includes a psychiatrist, psychiatric fellows and licensed clinical social workers.

Our study affirms the importance of capturing patient-reported HRQoL which will help identify and validate other potential clinical recommendations and interventions that can improve patient QoL over the course of treatment. Increased gynecologic cancer patient interaction with social workers and psychologists was predictive of improved patient functional well-being QoL from the pre-treatment to on-treatment period. Further studies of additional supportive psychosocial referrals and interventions for gynecologic cancer patients receiving radiotherapy are warranted to assess for causal impact.
